# Neuroimaging, cognition, light and circadian rhythms

**DOI:** 10.3389/fnsys.2014.00126

**Published:** 2014-07-08

**Authors:** Giulia Gaggioni, Pierre Maquet, Christina Schmidt, Derk-Jan Dijk, Gilles Vandewalle

**Affiliations:** ^1^Cyclotron Research Centre, University of LiègeLiège, Belgium; ^2^Centre for Chronobiology, Psychiatric Hospital of the University of BaselBasel, Switzerland; ^3^Surrey Sleep Research Centre, University of SurreyGuildford, UK

**Keywords:** sleep, circadian, light, non-image-forming, non-visual, fMRI, cognition, inter-individual differences in sleep-wake regulation

## Abstract

In humans, sleep and wakefulness and the associated cognitive processes are regulated through interactions between sleep homeostasis and the circadian system. Chronic disruption of sleep and circadian rhythmicity is common in our society and there is a need for a better understanding of the brain mechanisms regulating sleep, wakefulness and associated cognitive processes. This review summarizes recent investigations which provide first neural correlates of the combined influence of sleep homeostasis and circadian rhythmicity on cognitive brain activity. Markers of interindividual variations in sleep-wake regulation, such as chronotype and polymorphisms in sleep and clock genes, are associated with changes in cognitive brain responses in subcortical and cortical areas in response to manipulations of the sleep-wake cycle. This review also includes recent data showing that cognitive brain activity is regulated by light, which is a powerful modulator of cognition and alertness and also directly impacts sleep and circadian rhythmicity. The effect of light varied with age, psychiatric status, *PERIOD3* genotype and changes in sleep homeostasis and circadian phase. These data provide new insights into the contribution of demographic characteristics, the sleep-wake cycle, circadian rhythmicity and light to brain functioning.

## Introduction

Cognitive brain responses and performance vary between and within individuals. In recent years, there has been a growing interest in the contribution of circadian rhythmicity and sleep-wake regulation to the within and between subject variation.

Typically, wakefulness and its associated cognitive processes are maintained for 16 continuous hours before sleep is initiated for about 8 h. This sleep-wake alternation is regulated by two mechanisms: the circadian and the homeostatic processes (Borbély, 1982; Daan et al., 1984). There is evidence that the impact of the interaction between these two processes is not linear such that variations in performance and brain function are particularly pronounced when wakefulness is extended into the biological night, when the combined influence of circadian and homeostatic processes is particularly negative for cognition (Dijk et al., 1992; Wyatt et al., 1999, 2004; Cohen et al., 2010). Importantly, time-of-day variations in cognitive performance differ between individuals, and particularly during the biological night, suggesting differences in the interplay between circadian and homeostatic processes. Moreover, light has traditionally been related to the circadian clock, but also conveys a direct (exogenous) stimulating signal that impacts on alertness and cognition (Lockley et al., 2006; Chellappa et al., 2011). Furthermore, inter-individual differences in the sensitivity to the impact of light are also starting to emerge (Vandewalle et al., 2011a; Chellappa et al., 2012).

The aim of the present review is to summarize recent neuroimaging studies providing the first neural correlates of the endogenous and exogenous regulation of sleep, wakefulness and cognition. We first describe the basics of sleep/wakefulness regulation. Next, we present functional neuroimaging studies describing variations of subcortical and cortical cognitive brain activity, during a normal waking day and following acute sleep deprivation. To investigate interindividual differences in sleep-wake regulation, chronotype and a polymorphism in *PERIOD3* (*PER3*) were used. We then focus on the impact of light on cognitive brain activity, and its interaction with circadian phase and sleep need. In the last section, a plausible scenario of the brain mechanisms through which sleep homeostasis, circadian rhythmicity and light affect cognition is presented.

### Cognitive brain function is temporally organized by two interacting processes

More than 30 years ago, the two-process model by Borbély and colleagues (Borbély, 1982; Daan et al., 1984) conceptualized sleep-wake regulation, by the interaction of a circadian and a homeostatic process. Sleep homeostasis is characterized by an increase or dissipation of sleep pressure, as wakefulness extends or sleep progresses, respectively, and is almost exclusively dependent on sleep-wake behavior. The mechanisms underlying this hourglass-like process are still debated, but animal research suggests that it arises from a use-dependent local augmentation of sleep-promoting substances (adenosine (Basheer et al., 2004) and cytokines (Krueger, 2008)), from an increase in extracellular glutamate level (Dash et al., 2009), and/or from an experience-dependent increase of average brain synaptic strength, excitability and size during wakefulness (Vyazovskiy et al., 2008; Bushey et al., 2011). Other molecular markers of sleep loss have been identified in rodents (Franken and Dijk, 2009), while human polymorphisms have been associated with difference in sleep regulation [e.g., *PERIOD3 (PER3)* (Viola et al., 2007)*, Adenosine Deaminase (ADA)*,* Adenosin A2a receptor (ADORA2A), Brain Derived Neurotrophic Factor (BDNF), Catechol-O-Methyltransferase (COMT)*, *human leukocyte antigen (HLA)*, (Goel and Dinges, 2011), *dopamine transporter (DA)*, (Valomon et al., 2014), *ABCC9* (Allebrandt et al., 2013); for review see (Landolt, 2011)]. At the macroscopic scale, the electroencephalogram (EEG) provides the best established markers of sleep need and intensity: slow wave activity (SWA; 0.5–4 Hz) during Non-Rapid Eye Movement (NREM) sleep (Dijk et al., 1987, 1997), and theta activity (4–8 Hz) during wakefulness (Cajochen et al., 2002). Such increases are particularly marked over frontal EEG derivations, the frontal cortex being particularly sensitive to the sleep pressure (Cajochen et al., 1999a). Besides global increases, SWA changes are also detected locally in areas most implicated in the task previously performed during wakefulness (Kattler et al., 1994), likely reflecting synaptic changes (Huber et al., 2004; Hung et al., 2013).

Behaviorally, increased sleep pressure is associated with a deterioration of cognitive performance, a decrease in alertness and an increase in sleepiness (Dijk et al., 1992; Wyatt et al., 1999; Lo et al., 2012). However, cognitive performance and its associated brain activity do not linearly decrease with increasing amount of time spent awake. This shows that a second, circadian regulation process impinges on cognition. The circadian signal is defined as a near-24 h endogenous, self-sustained oscillator, which determines the timing of the rest-activity cycle and of most physiological processes in synchrony with the environmental light-dark cycle. It is controlled by the suprachiasmatic nucleus (SCN), located in the anterior hypothalamus, also known as the circadian master clock (Moore, 2007).The circadian signal increasingly promotes wakefulness during the day, opposing the progressive accumulation of sleep pressure. It reaches a maximum level, in the so called wake-maintenance zone, in the evening (typically between 8 PM and 10 PM for an 11 PM–7 AM habitual sleep episode), preventing us from falling asleep despite the high need for sleep (Strogatz et al., 1987; Dijk and Czeisler, 1994, 1995). Once passing into the biological night, the circadian signal turns into a sleep-promoting signal, which increasingly opposes the dissipation of homeostatic sleep pressure during sleep, allowing a consolidated 8 h sleep episode. Although still putative, a sense of this circadian sleep-promoting signal can be found in the regulation of REM sleep and sleep spindles, which are most prominent at the end of the night (Dijk and Czeisler, 1995).

In humans, core body temperature (CBT) circadian profile is probably the closest to the dynamics of the circadian signal promoting wake/sleep. CBT progressively increases during the day to peak in the evening (at around 10 PM), before initiating a progressive decrease until the end of the night (at around 6 AM) (Dijk and Czeisler, 1995). Other gold-standard markers of the circadian process are melatonin and cortisol levels (Czeisler et al., 1999). The onset of melatonin secretion, a hormone signaling the circadian night, coincides with the end of the wake-maintenance zone and CBT maximum. Melatonin secretion increases until 2–3 h prior to CBT minimum. The well-known increase in cortisol upon awaking is considered as a marker of the end of the putative sleep-promoting zone and, being activating, has been suggested to provide a gate for the transition between sleep and wakefulness (Czeisler and Gooley, 2007).

The interplay between the circadian and homeostatic processes not only determines sleepiness and alertness levels, but also affects higher order cognitive functions (Dijk et al., 1992). During a normal waking day, the increase in homeostatic sleep pressure and deterioration in brain activity are counteracted by the circadian alerting signal. However, when wakefulness is extended into the biological night, the circadian system no longer opposes the high need for sleep, and cognitive performance is jeopardized, most strongly at the end of the night when the circadian signal maximally favors sleep (Dijk and Archer, 2010). Following chronic sleep restriction, which is common nowadays, the circadian signal cannot efficiently oppose abnormally high sleep pressure and maintain adequate performance already during the day. In addition, if wakefulness is extended into the biological night following chronic sleep restriction, the negative impact of acute sleep deprivation on cognitive performance is exacerbated (Lo et al., 2012).

## Neural correlates of time-of-day changes in brain function

Ten years ago, a positron emission tomography (PET) study investigated changes in brain glucose metabolism between morning and evening acquisitions (Buysse et al., 2004). Compared to the morning, evening quiet wakefulness was associated with increased metabolism in hypothalamic and brainstem structures, putatively encompassing several sleep/wake or arousal promoting nuclei. Decreased metabolism was also found at the cortical level in the temporal and occipital lobes (Buysse et al., 2004). Yet, a more recent PET study suggested no significant difference in metabolism (glucose and oxygen consumption) between morning and evening measurements (Shannon et al., 2013). However, using resting state fMRI data, changes in functional connectivity between the medial temporal lobe (MTL) and the rest of the brain were detected between morning and evening measurements (Shannon et al., 2013). In the morning, bilateral MTL regions were mainly functionally connected to local areas, while their connectivity spread cortically in the evening, in a set of regions important for memory consolidation. Since these effects did not appear to be affected by the length of prior wakefulness (they were unchanged following sleep deprivation), the authors speculated that these changes may reflect aspects of memory consolidation recurring on a daily basis. Similarly to the numerous studies investigating the impact of sleep deprivation on cognitive brain function (for review see Chee and Chuah, 2008), the latter two studies were in fact not designed to disentangle the changes associated with circadian and sleep homeostatic processes, or their interaction. In addition, the conditions, in which subjects were maintained in between morning and evening PET and fMRI measures, were not carefully controlled for.

Critically for this review, there is evidence that there is a large inter-individual variability in the cognitive reaction to sleep loss. Some individuals are more resilient than others (Frey et al., 2004; Van Dongen et al., 2004; Viola et al., 2007), suggesting that additional factors affect the interplay of circadian and homeostatic processes. In the next two sections, we focus on three recent neuroimaging studies, which used known markers of interindividual differences in circadian and homeostatic sleep-wake regulation, and carefully controlled experimental conditions. These studies investigate interindividual variability in the temporal organization of cognitive brain function, during a normal sleep-wake cycle as well as after sleep deprivation.

## Chronotype and time-of-day influence on brain activity sustaining basic forms of attention and executive processes

Schmidt et al. (2009, 2012) used extreme chronotypes and their differences in sleep-wake regulation in order to investigate variations in brain activity. Differences in circadian timing preference are expressed in favorite periods of diurnal activity, such as working hours, and in specific sleep habits (Taillard et al., 2003), reflecting individual’s particular chronotype. Extreme morning types are located at one end of the continuum. They show a marked preference for waking up very early, and find it difficult to remain awake beyond their usual bedtime. At the opposite end, extreme evening types prefer to go to bed in the late hours of the night, and often find it extremely difficult to get up in the morning. Extreme chronotypes are “phase-shifted” according to their circadian rhythmicity, that is, the peaks and troughs of their physiological circadian markers (CBT, melatonin) occur earlier or later in relation to the external clock time (Kerkhof and Van Dongen, 1996; Duffy et al., 1999; Baehr et al., 2000; Bailey and Heitkemper, 2001; Duffy et al., 2001; Mongrain et al., 2004). Furthermore, chronotypical differences have also been observed in the phase relationship between sleep-wake cycle and underlying circadian rhythms (phase angle of entrainment) (Duffy et al., 1999; Baehr et al., 2000; Liu et al., 2000). However, the finding of phase-angle differences in chronotypes has not been systematically replicated (Bailey and Heitkemper, 2001; Mongrain et al., 2004). Accumulating evidence also suggests that chronotypes differ in their homeostatic sleep regulation. Morning types have been reported to show a faster build-up (Taillard et al., 2003) and dissipation rate of homeostatic sleep pressure. Likewise, morning types also tend to begin their sleep episode with higher SWA levels in anterior brain areas (Mongrain et al., 2006b). Chronotypes have thus been shown to differ in circadian and homeostatic sleep-wake regulatory processes, and constitute an appropriate tool to investigate the interaction of circadian and homeostatic processes under normally entrained day-night conditions.

**Figure 1:**
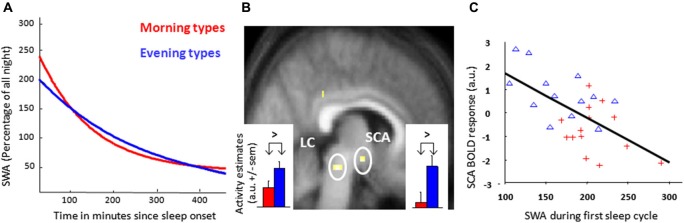
**Morning and evening chronotypes differ in their brain responses to an attentional task and SWA. (A)** Exponential decay function adjusted on relative SWA in sleep cycles (NREM sleep) measured from the central frontal derivation for all-night EEG of the night preceding the evening scan acquisition. **(B)** Increased task-related response in the dorsal pontine tegmentum and the anterior hypothalamus, compatible with the locus coeruleus (LC) and suprachiasmatic area (SCA) respectively, in evening as compared to morning chronotypes during the subjective evening for optimal sustained attention during the performance of a Psychomotor Vigilance Task (PVT). Corresponding activity estimates (arbitrary units—a.u. +/− sem) are displayed for event indicators of fast reaction times. **(C)** Regression analysis of the relation between estimated blood oxygen level-dependent (BOLD) responses during optimal task performance in the SCA region and the amount of SWA during the first sleep cycle in the preceding night (*r* = 0.54, *p* < 0.05, *n* = 27). Red crosses: morning types, blue triangles: evening types. [Copied with permission from Schmidt et al. (2009); Cajochen et al. (2010)].

In both papers of Schmidt et al. (2009, 2012), extreme morning and evening types underwent a morning and an evening fMRI session, which was timed according to unconstrained preferred sleep and wake times, respectively 1.5 h and 10.5 h after wake-up time. During the fMRI sessions, two successive tasks were administered: the psychomotor vigilance task (PVT; Dinges and Powell, 1985) and the Stroop task (Stroop, 1935). The PVT records reaction times (RT) to random occurrences of a simple visual cue and probes sustained attention, i.e., the ability to maintain attention over prolonged periods of time (Dinges and Powell, 1985) which is considered as a fundamental form of attention onto which many other cognitive processes build (Raz and Buhle, 2006). The PVT has been repeatedly used to show circadian and sleep homeostatic influences on cognition (Lim and Dinges, 2008). Schmidt et al. (2009) focused on two PVT measures: global alertness, corresponding to trials with intermediate RT, and optimal alertness, associated with fastest RTs, phasically occurring when the participant is able to recruit attentional resources above and beyond the normal level set by global alertness. Only small differences between chronotypes were observed in the morning session, when homeostatic sleep pressure is low. By contrast, in the evening session, when sleep pressure is higher and the circadian signal strongly promotes wakefulness, global alertness was associated with increased thalamic responses in morning as compared to evening types. The observed response was located in a dorsomedial part of the thalamus compatible with the anterior pulvinar. Interestingly, the pulvinar has been showed to actively regulate cortical activity based on attention demand (Saalmann et al., 2012). Pulvinar activation has also been related to arousal level following total sleep deprivation (Portas et al., 1998), noradrenaline administration (Coull et al., 2004), as well as during and following light exposure as an external activating factor (Vandewalle et al., 2006). The result of Schmidt et al. (2009) suggests therefore that, with higher homeostatic sleep pressure and compared to evening types, morning types are under more demanding condition and recruit relatively more the pulvinar for maintaining a normal global alertness level. In addition, maintaining optimal alertness in the subjective evening was associated with larger responses, in evening compared to morning chronotypes, in a brainstem region compatible with the locus coeruleus (LC; Schmidt et al., 2010), and the suprachiasmatic area (SCA), i.e., an anterior hypothalamus region that encompassed the SCN (Figure 1B). The LC is the major source of norepinephrine and has widespread thalamic and cortical connections, so that it can potentially modulate higher-order cognitive functions (Aston-Jones, 2005). LC and SCN are two connected structures involved in the generation of the circadian arousal signal, which could regulate cognitive output during a waking day (Aston-Jones, 2005). Thus, the known improved cognitive ability of evening chronotypes towards the end of a normal waking day may result from their ability to recruit these interacting subcortical structures above normal levels.

When analysing polysomnographic data, SWA of the first NREM cycle was higher and dissipated faster in the course of the night in morning types, in agreement with previous findings (Mongrain et al., 2006b; Figure 1A). An independent regression analysis revealed that SCA activity related to optimal task performance was inversely proportional to SWA of the first NREM cycle (Figure 1C). This observation suggests a negative relation between homeostatic sleep pressure and response associated with optimal alertness during a PVT within the SCA, putatively encompassing the circadian master clock. Interestingly, data obtained in rodents similarly point to an impact of homeostatic sleep pressure markers on electrical activity within the SCN (Deboer et al., 2003, 2007). Globally, the results suggest that evening types are more able to recruit arousal-promoting brain structures to maintain optimal alertness, even with increasing homeostatic sleep pressure. It may be assumed that performance of morning types would deteriorate in the evening, through a negative impact of sleep pressure on the master circadian clock that is not compensated by the increase in thalamic activation associated with global alertness. Conversely, it may be through a decreased ability of anterior hypothalamic activity to counteract sleep pressure that morning types undergo greater performance decrement.

The next logical question is whether sleep-wake regulatory processes similarly or differently affect more demanding executive processing. Schmidt et al. (2012) then turned to the second task of their protocol, the Stroop task, which probes interference by challenging continuous control over conflicting information. In this color-word task, subjects have to indicate as quickly as possible the color in which a word is printed while ignoring its meaning (e.g., incongruent item: word “blue” printed in red, congruent item: word “blue” printed in blue). By comparing congruent to incongruent items, the task allows the isolation of brain activity linked to cognitive interference. From the subjective morning to the subjective evening, evening types maintained or even increased interference-related responses in a set of brain areas playing a pivotal role in successful inhibitory functioning, whereas morning types presented decreased responses under the same conditions (Schmidt et al., 2012). Furthermore, in the evening, a regression showed that interference-related fMRI activity in the posterior part of the hypothalamus was negatively related to SWA of the first sleep cycle for morning types, whereas no significant correlation was found in evening types. This hypothalamic cluster was more posterior than in the PVT study, and was compatible with the lateral hypothalamus (LH), site of orexin (ORX) and melanin-concentrating hormone (MCH; Adamantidis and de Lecea, 2008), close to the location showing decreased gray matter concentration in narcoleptic patients (Draganski et al., 2002). These findings suggest that, in evening types, promotion and maintenance of appropriate cognitive interference abilities at the cortical level depend on posterior hypothalamus activity in the evening, when homeostatic sleep pressure is higher. In morning types, a relative weakness in the transmission of alerting signals from subcortical structures, e.g., the posterior hypothalamus, to the cortex could be the reason of decreased activity in interference-related brain structures from the morning to the evening hours.

## *PERIOD3* genotypes, time-of-day and sleep-loss influences on cognitive brain responses

The following study we present used *PER3* genotype to investigate interindividual differences in the negative effect of sleep loss on brain activity. A primate-specific variable number tandem repeat (VNTR) polymorphism (4 or 5 repeats) in the coding region of the clock gene *PER3* is associated with individual preference of waking activity and sleep (Archer et al., 2003; Lázár et al., 2012), with *PER3*^5/5^ showing morning preferences and* PER3*^4/4^ evening preferences. Approximately 10% of the population is homozygous for the 5-repeat (*PER3*^5/5^), whereas 50% present the 4-repeat (*PER3*^4/4^). Although the two genotypes do not differ in circadian phase, *PER3*^5/5^ seem to have a more rigid circadian control (Archer et al., 2008). Regarding sleep characteristics, *PER3*^5/5^ have shorter sleep latency, more slow wave sleep (SWS) and more SWA, particularly in the first part of the night, both following a normal waking day or prolonged wakefulness, a profile similar to the one observed in morning types (Viola et al., 2007). During sleep deprivation, analysis of the waking EEG reveals a more rapid increase of theta/alpha activity and more frequent slow eye movements, a marker of inattention and drowsiness (Cajochen et al., 1999b) in *PER3*^5/5^, compared to* PER3*^4/4^ participants. Furthermore, during the recovery night from sleep deprivation, the compensatory increase in SWS leads to a stronger suppression of REM sleep in *PER3*^5/5^ (Viola et al., 2007), indicating a possible homeostatic regulation of SWS at the expense of REM sleep (Dijk and Archer, 2010). To summarize, both genotypes have an identical circadian oscillator but differ in the homeostatic regulation of sleep, with* PER3*^5/5^ having an accelerated build-up of sleep pressure (Dijk and Archer, 2010). These data have been in part replicated in a nap protocol (Maire et al., 2014b) and in older people (Viola et al., 2012).

**Figure 2:**
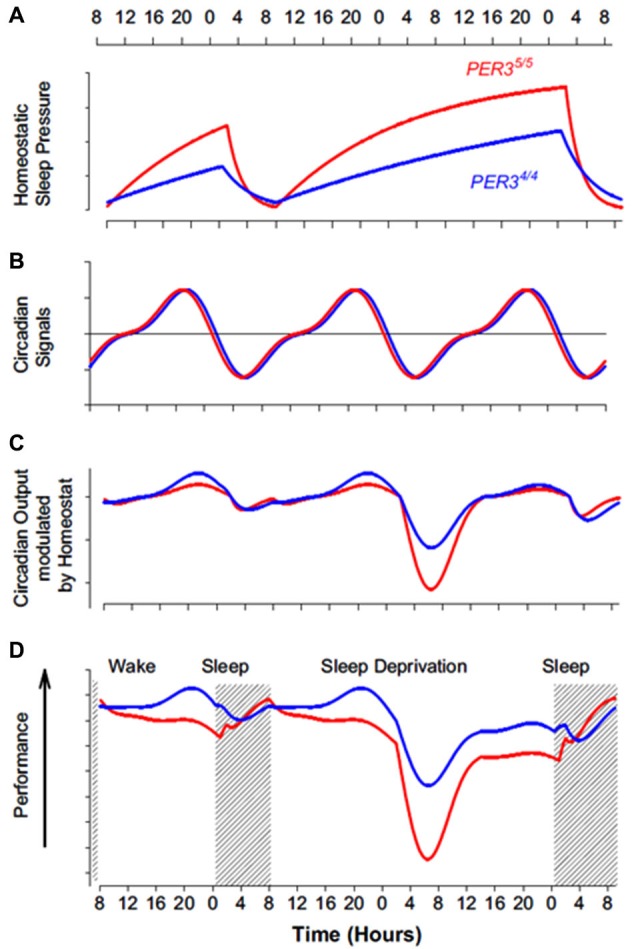
**Impact of the interaction between homeostatic sleep pressure and circadian process on cognitive performance in *PER3*^4/4^ (blue line) and *PER3*^5/5^ (red line) individuals**. **(A)** As indicated by slow wave activity (SWA) measure, *PER3*^5/5^ have a faster build-up during wakefulness and a quicker dissipation during sleep of homeostatic sleep pressure (based on data from Viola et al. (2007)). **(B)** Regarding circadian phase, *PER3*^4/4^ and *PER3*^5/5^ do not appear to differ, as indicated by melatonin, cortisol, and *PER3* mRNA measures (based on data from Viola et al. (2007)). Note that the circadian signal increasingly promotes wakefulness during the day (positive value, above horizontal line) and increasingly promotes sleep during the night (negative value, below horizontal line). **(C)** Theoretical modulation of the circadian signal by homeostatic sleep pressure in both *PER3* genotypes. The difference in homeostatic sleep pressure results in a limited difference in the output of this interaction during a normal waking day. The output of the interaction affects much more negatively wakefulness of *PER3*^5/5^ in the absence of overnight sleep, particularly in the early morning hours when the circadian system maximally promotes sleep. **(D)** Composite measures of performance in both *PER3* genotypes, based on extended neurophychological test batteries (Viola et al., 2007). Performance profile closely follow theoretical interaction between circadian and sleep homeostasis processes depicted in **C**. This model could speculatively be applied to extremes morning and evening chronotypes, which also differ in term of homeostatic sleep pressure build up (Mongrain and Dumont, 2007) (but also they differ sometimes in term of circadian phase angle with sleep (Mongrain et al., 2006a)). [Copied with permission from Dijk and Archer (2010)].

Regarding the impact of sleep deprivation on cognition, there also seem to be important divergences between genotypes. While performance remains similar between genotypes during a normal waking day, with a slightly larger decrement in *PER3*^5/5^, during early-morning hours following acute sleep loss, *PER3*^5/5^ have a worse decline in cognitive performance, particular so for executive tasks (Viola et al., 2007; Groeger et al., 2008). Furthermore, it has been shown that time-on-task decrement in performance observed during the realization of a PVT was also *PER3* genotype-dependent, with worse decrement for *PER3*^5/5^ (Maire et al., 2014a). A recent simple model considered performance as a result of a non-linear interaction between the circadian and homeostatic signals, and matched differences between *PER3* genotypes (Dijk and Archer, 2010). The non-linearity of this interaction can be particularly sensed in the early morning hours, when the circadian sleep-promoting signal amplifies the differences in homeostatic sleep pressure, such that performance deteriorates disproportionally in *PER3*^5/5^ individuals (Dijk and Archer, 2010; Figure 2). The latter model also explains why little behavioral differences were found between *PER3* genotypes that were allowed to sleep in the second half of the night during a sleep restriction protocol (Goel et al., 2009) (i.e., performance was not assessed during the second part of the night, when *PER3*-dependent effect are more pronounced).

Vandewalle et al. (2009a) studied normal volunteers prospectively recruited on their *PER3* genotype in an fMRI study. Each subject participated in two experimental segments, separated by at least 1 week, which were identical except for the presence or absence of sleep between the evening and morning fMRI sessions. The evening fMRI acquisition was scheduled 2 h before habitual bedtime, i.e., within the wake-maintenance zone, while the morning fMRI session was 1.5 h after wake time, after the putative sleep-maintenance zone. Thus, the morning and evening sessions differed with respect to both time awake and circadian phase, while morning sessions were scheduled at the same circadian phase and differed only for previous amount of time wake. In each session, participants performed an auditory working memory 3-back task (Cohen et al., 1997) in two consecutive recordings, once in darkness and once while exposed to light. Only the first recording will be considered here. The second will be summarized in the following section on the impact of light.

**Figure 3:**
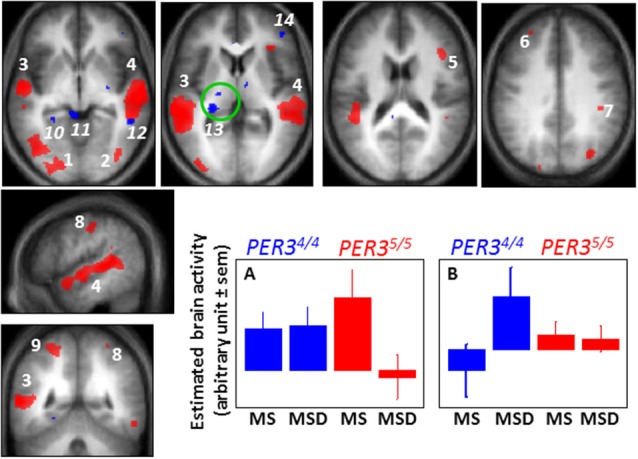
**Difference between *PER3*^4/4^ and *PER3*^5/5^ individuals in the sleep loss-induced changed in brain responses to a working memory task**. When comparing brain responses to an auditory 3-back task in the morning after a night of sleep (MS 1.5 h of wakefulness) and in the morning after a night of sleep deprivation (MSD 25 h of wakefulness), *PER3*^5/5^ individuals undergo marked decreases in activation in several brain areas of the occipital (1, 2) and temporal (3, 4) cortices, and of the dorsolateral prefrontal (5, 6) and parietal cortex (7–9), while *PER3*^4/4^ individuals maintain brain responses in these areas (and do not present significant decreased activations in any brain regions). A representative profile of this brain activity change is displayed in panel **A** (similar profiles were observed for red areas 1–9). In contrast, when comparing the same sessions, *PER3*^4/4^ individuals present increased activation (blue) in the parahippocampus (10), superior colliculus (11), temporal cortex (12), pulvinar (13), and ventrolateral prefrontal cortex (14), while no increased activation is observed in these regions in *PER3*^5/5^ (and in any other brain regions). A representative profile of this brain activity change is displayed in panel **B** (similar profiles were observed for blue areas 10–14). A significant negative association was found between overnight change in brain response in the pulvinar (green circle) and self-reported daytime propensity to fall asleep in everyday life across all the subjects of the study (irrespective of genotype), further suggesting a central role for the pulvinar in wakefulness regulation. [Adapted with permission from Vandewalle et al. (2009a)].

Analyses first focused on changes across a normal waking day and revealed that, in the evening relative to the morning session after sleep, *PER3*^5/5^ participants showed reduced activation in the posterior dorsolateral prefrontal cortex (DLPFC) implicated in higher executive control (Koechlin et al., 2003), while *PER3*^4/4^ did not show any significant changes. Comparing both morning sessions (low vs. high homeostatic sleep pressure) revealed an apparent double dissociation between the genotypes (Figure 3). *PER3*^5/5^ volunteers showed decreased activations following sleep loss in a widespread set of areas involved in the ongoing task (Cohen et al., 1997; Collette et al., 2005, 2006), including temporal, parietal and occipital areas, and again bilaterally in the DLPFC. By contrast, *PER3*^4/4^ participants recruited supplementary brain areas to perform the task in the ventro-lateral prefrontal cortex (VLPFC), temporal cortex, as well as a thalamic region compatible with the pulvinar. Comparisons between the morning session after sleep deprivation and the evening wake-maintenance zone session were then computed (large differences in the sleep/wake promoting circadian signal vs. intermediate differences in sleep pressure). Analyses revealed again increased compensatory activations in *PER3*^4/4^ and decreased activations in *PER3*^5/5^in the same cortical and subcortical regions than those observed when comparing morning sessions. Importantly, these activation profiles were even more pronounced than when comparing morning after sleep and morning after sleep deprivation sessions, especially in *PER3*^5/5^ individuals. If sleep homeostasis was primarily responsible for differences observed, then differences should be reduced when comparing the morning session after sleep deprivation to the evening session after a normal waking day. Likewise, if the circadian signal was solely responsible for changes in cognitive brain responses, then a difference should only be present when comparing morning to evening sessions.

Taken together these data and those of Schmidt et al. (2009, 2010) confirm that the daily temporal organization of executive brain activity depends on the endogenous mechanisms regulating sleep and wakefulness, i.e., the interplay between sleep homeostasis and the circadian clock. A question, then asked by Vandewalle et al. (2011a) was how this daily temporal organization was affected in the presence of an exogenous factor impinging on sleep and wakefulness: light exposure. After a brief reminder of previous findings on the effect of light on brain function, we will summarize results obtained by investigating groups differing with respect to *PER3* genotype, age or psychiatric status.

## Light stimulates cognitive brain function and directly affects sleep and wakefulness

Light is necessary for image formation by the visual system, but is also essential for the regulation of numerous circadian, neuroendocrine, and neurobehavioral non-image-forming (NIF) functions, including the direct improvement of alertness and performance (Vandewalle et al., 2009b; Hatori and Panda, 2010; Schmidt et al., 2011; Bailes and Lucas, 2013). These NIF effects of light are mediated in part by a retinal photoreception system, which is distinct from the classical visual system. In addition to rods and cones, the NIF photoreception system recruits a novel class of photoreceptors, which consists in intrinsically photosensitive retinal ganglion cells (ipRGC) expressing the photopigment melanopsin and maximally sensitivity to blue light (ca 480 nm) (Berson et al., 2002; Dacey et al., 2005; Hatori and Panda, 2010; Schmidt et al., 2011; Bailes and Lucas, 2013). The ipRGCs play accessory visual functions (Ecker et al., 2010; Brown et al., 2012), but are deeply implicated in the NIF functions of light, which have therefore a sensitivity shifted toward shorter wavelength blue light. IpRGC constitutes the only channel though which light affects NIF functions (Guler et al., 2008); however, inputs from rods and cones are necessary to observe a complete response (Ruby et al., 2002). Melanopsin-expressing ipRGCs project to various brain structures, including hypothalamic, thalamic, striatal, brainstem and limbic structures (Hattar et al., 2006; Ecker et al., 2010). Importantly, ipRGCs have direct projections to the SCN. These widespread and numerous projections are an essential feature of the brain mechanisms, through which light can exert a potent and diverse impact on NIF functions.

**Figure 4:**
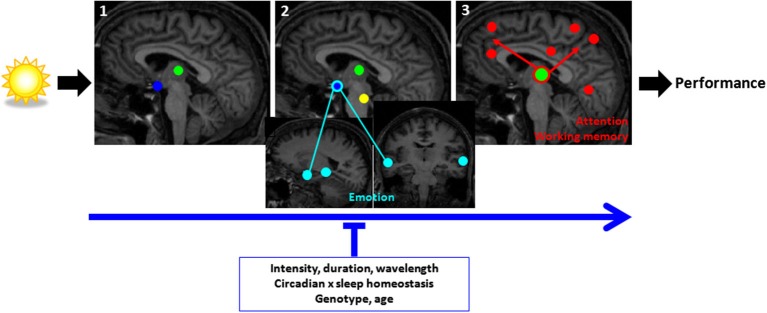
**Schematic representation of the brain mechanisms involved in the non-image-forming impact of light on cognitive brain responses**. **(1)** Responses at light onset are found within the hypothalamus (blue) and pulvinar (green) (and amygdala and hippocampus, not shown); **(2)** within the first seconds of the exposure, responses are found mainly in subcortical and cortical structures involved in alertness regulation (hypothalamus, brainstem (yellow), pulvinar); **(3)** late responses are detected at the cortical level in areas involved in the ongoing cognitive process and can subsequently affect performance. For attention/working memory/executive task (red) a network of areas around the pulvinar and including prefrontal and parietal areas appear to mediate the impact of light on alertness and cognition. For emotional responses to vocal stimuli (light blue), the network involves the hypothalamus, amygdala, and voice-sensitive area of the temporal cortex. Light seems to have a swifter impact on emotional cortical responses than attentional/working memory/executive responses. The impact of light is stronger with higher intensity, longer duration, and shorter wavelength (blue) light exposures. Time of day and the associated changes in the interaction between circadian and sleep homeostasis signals and *PERIOD3* genotype modulate the impact of light. [Adapted with permission from Vandewalle et al. (2009b)].

Light can affect sleep, wakefulness and cognition indirectly, via its synchronizing/phase-shifting effects on the circadian clock. Critically, light also conveys a direct stimulating signal that affects sleep homeostasis (Cajochen et al., 1992; Altimus et al., 2008; Tsai et al., 2009; Chellappa et al., 2013), increases alertness and cognitive performance (Cajochen et al., 2005; Rahman et al., 2014), including during sleep inertia (Santhi et al., 2013). A series of neuroimaging studies investigated the brain mechanisms involved in the impact of light on cognition, using simple attentional task (oddball paradigm) (Vandewalle et al., 2006), more complex working memory task (n-back) (Vandewalle et al., 2007a, 2011b), and emotional tasks (Vandewalle et al., 2010). In accordance with animal research, results of these studies are compatible with a scenario in which light would first influence subcortical structures involved in arousal regulation, before significantly affecting the cortical areas involved in the ongoing cognitive process (Vandewalle et al., 2009b; Vandewalle and Dijk, 2013; Figure 4). These subcortical structures include hypothalamus nuclei, possibly the SCN, the ventrolateral preoptic area, the dorsomedian hypothalamus, and/or the paraventricular nucleus of the hypothalamus (PVNH). The brainstem also appears to play a central role, within the LC or in other nuclei of the ascending activating system, while similarly, the pulvinar is repeatedly affected. Light impact on the latter two structures could greatly affect information flow within the cortex. If sufficient, light subcortical impact would then significantly affect the cortical area recruited for the ongoing cognitive process. Behavioral measures would only be significantly affected after prolonged light exposure, either because the light impact on cortical structures requires time to be transferred into behavior, or because behavioral measures are less sensitive than neuroimaging techniques, or probably both. Although photoreceptors are not as accessible in human as in animal models (e.g., genetic modifications are not possible), two recent studies provided compelling evidence that melanopsin-expressing ipRGCs were mediating the impact of light on cognitive brain responses (Vandewalle et al., 2013; Chellappa et al., 2014).

## Effects of light on cognitive brain responses depend on endogenous processes affecting sleep and wakefulness

Aging is associated with important changes in the regulation of sleep and wakefulness. Briefly, sleep becomes more fragmented and the amount of SWS decreases, suggesting a less restorative sleep but a shallower build-up of sleep need (Klerman and Dijk, 2008; Carrier et al., 2009). The amplitude of the circadian signal also appears to be reduced in aging, as indicated by the reduced detrimental effect of night-sleep loss at the behavioral level, but also by earlier awakening during sleep (Daneault et al., 2013). Daneault et al. (2014) recently reported that, even if still present, the impact of light on brain responses was reduced in healthy older individuals (>60 y.o.), compared to younger individuals (<30 y.o.), when investigated after habitual sleep time (and therefore after the wake maintenance zone). Reduced impacts of light were observed notably within the insula, prefrontal cortex, amygdala, tegmentum and thalamus, which are key structures in the regulation of alertness and cognition.

In another study, patients suffering from Seasonal Affective Disorder (SAD—winter depression) were shown to present abnormal responses to emotional stimulation within the posterior hypothalamus, in a region compatible with the ORX/MCH LH or with the PVNH (Vandewalle et al., 2011b). Similar to aging, SAD is characterized by changes in the regulation of sleep and wakefulness (Cajochen et al., 2000), but with patients sleeping more and showing decreased motivation and mood.

The latter studies on SAD and aging could indicate that endogenous changes in sleep-wake regulation modify the impact of light on cognitive brain activity, or alternatively that changes in the impact of light contribute to changes in sleep-wake regulation. However, these studies did not include measures repeated over the 24 h day, so that no inference with respect to sleep homeostasis or circadian processes can be made. For instance, older individuals recruited more brain areas to perform the task independent of the light condition (Hedden and Gabrieli, 2004). The diminished impact of light is maybe due to the fact that older individuals were compensating for task difficulty and could not be helped as efficiently by light.

As already mentioned, Vandewalle et al. (2011a) also collected data including the *PER3* genotype and light exposure. The same participants were exposed to alternating blue and green light while performing a 3-back task. Light wavelengths were chosen to maximally stimulate the NIF photoreception system (blue) or the classical photopic system (green). Brain responses to the task under blue and green light were compared in the morning following sleep, in the evening wake-maintenance zone, and in the morning following sleep deprivation.

Results indicated that, in the morning shortly after sleep, blue light significantly enhanced brain responses to the task in prefrontal and parietal areas, as compared to green light (Vandewalle et al., 2011a). These blue light effects were only found in *PER3*^4/4^ individuals. Surprisingly, no differential impacts of light wavelength were found in the evening wake-maintenance zone, indicating a relative decrease of the impact of light in that part of the circadian cycle. Finally, in the morning session after sleep deprivation, blue light significantly increased task-related brain activity. This blue light effect was observed again in the prefrontal and parietal cortices, but also in other areas, including the insula and the pulvinar (Vandewalle et al., 2011a). Importantly, in the morning after sleep deprivation, these effects of blue light were only observed in *PER3*^5/5^.

## A putative scenario of the brain mechanisms involved in the interplay between cognition, sleep-wake regulation and light

Collectively, these neuroimaging studies investigating interindividual differences allow for a scenario speculating about the effects of homeostatic sleep pressure, the circadian signal and of light on cognition-related brain activity. It appears that the anterior hypothalamus, in a region compatible with the SCA, but also the ORX/MCH posterior LH, may constitute sites through which circadian and homeostatic processes interact (Schmidt et al., 2009, 2012) for the regulation of cognitive brain activity. Based on the data, one could also consider the hypothalamus as one of the first structures affected by light, within the SCA (Perrin et al., 2004), but also possibly in other nuclei such as the PVNH, dorsomedial hypothalamus (DMH) or LH in the context of an emotional task (Vandewalle et al., 2010, 2011b). Importantly, the hypothalamus, and particularly the SCN, is indirectly connected with the LC, a region of the brainstem that is the main source of norepinephrine (Aston-Jones, 2005), and is likely to be the brainstem region influenced by light in a nonvisual context (Vandewalle et al., 2007b). Both structures are highly implicated in the circadian regulation of sleep and wakefulness and have multiple connections to other relevant brain areas, including the thalamus, and cortex for the LC. Thus, the hypothalamus and LC could be the subcortical core that regulates the circadian alerting signal and the stimulating impact of light.

For more demanding cognitive challenges (e.g., late evening hours, sleep loss, higher order executive tasks), cortical regions seem to enter into play. When testing brain responses in the evening, both morning chronotypes and *PER3^5/5^* individuals were unable to maintain stable brain responses to cognitive inhibition or working memory tasks (Vandewalle et al., 2009a; Schmidt et al., 2012). In addition, when testing working memory in the morning following sleep deprivation, the recruitment of the lateral prefrontal cortex (LPFC) appears as a key factor for the maintenance of brain responses. *PER3*^5/5^ are unable to maintain activation in DLPFC (Vandewalle et al., 2009a). By contrast, the recruitment of the VLPFC by* PER3*^4/4^ under sleep deprivation may reflect a compensatory switch to a more appropriate cognitive strategy (Vandewalle et al., 2009a). The frontal lobe plays a major role in executive function and, according to a model, the VLPFC is important for cognitive control and is involved in complex neurobehavioral processes (Koechlin et al., 2003).

The pulvinar, which was more activated following sleep deprivation in *PER3^4/4^* individuals, appears also to play a central role in the ability to face sleep loss and circadian challenges, and may constitute a further subcortical site through which circadian and sleep homeostasis interaction affects cognition and alertness (Aston-Jones, 2005). This assumption is strengthened by supplemental analyses indicating a significant negative association between overnight change in task-related pulvinar brain responses and daytime propensity to fall asleep in everyday life across all the subjects of the study (i.e., irrespective of genotype) (Vandewalle et al., 2009a).

But how does the difference in the impact of light fit in this picture? Differences in the endogenous drive for wakefulness, or in compensatory mechanisms already in place, stand as a likely explanation. The combination of sleep loss and adverse circadian phase induces major reductions of activations across all parts of the cortex in *PER3^5/5^* individuals. Blue light appears to be effective in “rescuing” brain responses under these adverse circumstances. On the other hand, *PER3*^4/4^ individuals are able to trigger endogenous compensatory brain mechanisms that maintain brain responses in the morning after a night without sleep, and blue light is less beneficial to them. The nonvisual impact of light would therefore provide more benefits to the genotype that is not able to maintain brain responses endogenously and is most challenged by the circadian and sleep homeostatic conditions. This would be the reason why a relative decrease in the stimulating impact of light was detected in both genotypes in the wake-maintenance zone, when the endogenous drive for wakefulness is maximum. In addition, *PER3*^5/5^ individuals are more likely to be morning chronotypes and prefer to be active in the morning hours (Archer et al., 2003), so that in the morning following sleep they would be in optimal endogenous conditions to perform, and could not benefit as much from an external light stimulation. *PER3*^4/4^ individuals, which represent 45–50% of the general population and are more likely to be evening chronotypes, would benefit more from light in the morning after a night of sleep. This hypothesis is in agreement with previous studies, which were carried out in the morning (after a normal night of sleep), and found a significant impact of light on brain responses in non-genotyped samples (Vandewalle et al., 2006, 2007a,b). Again the pulvinar, which was affected by blue light following sleep deprivation in *PER3*^5/5^, also constitutes a possible interface between light impact and cortical cognitive brain responses.

## Conclusion and perspective

**Figure 5:**
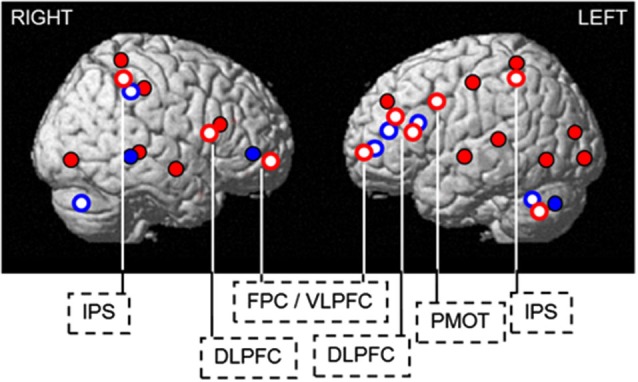
**Endogenous and exogenous regulation of sleep and wakefulness affects cognitive brain responses through overlapping pathways**. Blue light increases cognitive brain responses in regions showing decreased activation (*PER3*^5/5^) or compensatory recruitment (*PER3*^4/4^) in darkness, following sleep loss. Blue solid: compensatory increase in activation in the morning hours after 25 h of wakefulness in *PER3*^4/4^, found in the ventrolateral prefrontal cortex, temporal cortex, cerebellum, and thalamus (not shown). Red solid: decreases in activation in the morning hours after 25 h of wakefulness in *PER3*^5/5^, observed in the occipital, temporal, parietal, and lateral prefrontal cortices. Red open: blue light-induced increase in activations after 25 h of wakefulness in *PER3*^5/5^(thalamus not shown). Blue open: blue light-induced increase in activity after 1.5 h of wakefulness in *PER3*^4/4^. DLPFC = dorsolateral prefrontal cortex; FPC / VLPFC = frontopolar / ventrolateral prefrontal cortex; IPS = intraparietal sulcus; PMOT = premotor cortex. [Copied with permission from Vandewalle et al. (2011a)].

It is maybe remarkable that endogenous and exogenous mechanisms, regulating sleep and wakefulness, affect cognitive brain responses through at least partially overlapping pathways. These overlaying pathways become more obvious when overlaying differences in brain responses observed between *PER3* genotypes with and without light exposure (Figure 5). Thus we can gain insight into cognition regulation by manipulating wakefulness either endogenously or exogenously.

Overall, an “inverted U shape” profile, which would differ between individuals regarding chronotype or vulnerability to sleep loss, could fit with the results summarized in this review. In all individuals, accumulation of homeostatic sleep pressure would initially be associated with activation of arousal-related thalamic regions, and cortical areas, including prefrontal areas when higher cognitive processes are involved. The thalamus was indeed activated in morning types in the evening for a simple cognitive challenge. When the challenge becomes too adverse, brain responses are not maintained in these regions. This decreased-activation process begins at a different moment, depending on circadian and homeostatic characteristics of an individual. Morning chronotypes and *PER3*^5/5^ seem to have a more rigid circadian control and perform better in the morning. Because of a faster homeostatic sleep pressure build-up, their cognitive resources would undergo a faster decrement, and they would benefit earlier of endogenous compensation, but suffer also from an earlier failure of these compensatory mechanisms. Instead, in the morning following a night without sleep, *PER3*^4/4^, and putatively evening chronotypes, would engage compensation later and would therefore be able to maintain cognitive brain responses better under more adverse conditions, i.e., morning hours following sleep loss. Light would help more efficiently those individuals that are far from the apex of the inverted U-shape. This would also be valid for older individuals, which are already compensating for an important cognitive challenge, and would benefit as much from light.

The scheme remains highly speculative and has to be further explored by subjecting extreme chronotypes or older individuals to stringent sleep deprivation protocols, with or without light, or by increasing the sampling rate of neuroimaging assessment, under constant routine or forced desynchronize protocol, and particularly around critical time point such as the wake and sleep maintenance zone.

## Conflict of interest statement

The authors declare that the research was conducted in the absence of any commercial or financial relationships that could be construed as a potential conflict of interest.
